# Distinct Oceanic Microbiomes From Viruses to Protists Located Near the Antarctic Circumpolar Current

**DOI:** 10.3389/fmicb.2018.01474

**Published:** 2018-07-17

**Authors:** Flavia Flaviani, Declan C. Schroeder, Karen Lebret, Cecilia Balestreri, Andrea C. Highfield, Joanna L. Schroeder, Sally E. Thorpe, Karen Moore, Konrad Pasckiewicz, Maya C. Pfaff, Edward P. Rybicki

**Affiliations:** ^1^Biopharming Research Unit, Department of Molecular and Cell Biology, University of Cape Town, Cape Town, South Africa; ^2^Marine Biological Association of the United Kingdom, Citadel Hill, Plymouth, United Kingdom; ^3^School of Biological Sciences, University of Reading, Reading, United Kingdom; ^4^College of Veterinary Medicine, University of Minnesota Twin Cities, Minneapolis, MN, United States; ^5^Limnology, Department of Ecology and Genetics, Uppsala University, Uppsala, Sweden; ^6^British Antarctic Survey, Natural Environment Research Council, Cambridge, United Kingdom; ^7^Exeter Sequencing Service, Biosciences, University of Exeter, Exeter, United Kingdom; ^8^Department of Environmental Affairs, Oceans and Coasts, Cape Town, South Africa

**Keywords:** Antarctic Polar Front, marine microbes, eDNA, meDNA, viruses, microbiome

## Abstract

Microbes occupy diverse ecological niches and only through recent advances in next generation sequencing technologies have the true microbial diversity been revealed. Furthermore, lack of perceivable marine barriers to genetic dispersal (i.e., mountains or islands) has allowed the speculation that organisms that can be easily transported by currents and therefore proliferate everywhere. That said, ocean currents are now commonly being recognized as barriers for microbial dispersal. Here we analyzed samples collected from a total of six stations, four located in the Indian Ocean, and two in the Southern Ocean. Amplicon sequencing was used to characterize both prokaryotic and eukaryotic plankton communities, while shotgun sequencing was used for the combined environmental DNA (eDNA), microbial eDNA (meDNA), and viral fractions. We found that Cyanobacteria dominated the prokaryotic component in the South-West Indian Ocean, while γ-Proteobacteria dominated the South-East Indian Ocean. A combination of γ- and α-Proteobacteria dominated the Southern Ocean. Alveolates dominated almost exclusively the eukaryotic component, with variation in the ratio of Protoalveolata and Dinoflagellata depending on station. However, an increase in haptophyte relative abundance was observed in the Southern Ocean. Similarly, the viral fraction was dominated by members of the order *Caudovirales* across all stations; however, a higher presence of nucleocytoplasmic large DNA viruses (mainly chloroviruses and mimiviruses) was observed in the Southern Ocean. To our knowledge, this is the first that a statistical difference in the microbiome (from viruses to protists) between the subtropical Indian and Southern Oceans. We also show that not all phylotypes can be found everywhere, and that meDNA is not a suitable resource for monitoring aquatic microbial diversity.

## Introduction

Microbes constitute more than 90% of oceanic biomass (Suttle, 2007). With more than 70% of the Earth’s surface covered by ocean, they drive almost half of the global net primary production (Azam et al., 1983; Cho and Azam, 1990; Field, 1998). Despite being so abundant, we have yet to uncover their full significance in ocean processes. This is largely due to the fact that the majority of microorganisms cannot be grown in the laboratory (Handelsman, 2004) and are challenging to identify morphologically. However, thanks to the developments in sequencing technologies, we are now unveiling marine microbial diversity without the need for cultivation (Loman et al., 2012; Flaviani et al., 2017). Nevertheless, the majority of sequences coming from marine (Sogin et al., 2006; Brum et al., 2013), soil (Roesch et al., 2007), or human gut (Turnbaugh et al., 2009) microbiome studies still remain largely uncharacterized.

Over the past 15 years, various attempts have been made to explore the microbiomes of the world’s oceans: these including expeditions such as the Global Ocean Sampling (2003–2010) (Rusch et al., 2007), Tara Ocean Expedition (2009–2012) (Sunagawa et al., 2015), Malaspina, 2010 (Laursen, 2011), or various census programs such as the Earth Microbiome program (Gilbert et al., 2011) and the Micro B3 which lead the Ocean Sampling Day (Kopf et al., 2015). However, microbial communities are rarely dispersed uniformly in both time and space (Deacon, 1982; Cao et al., 2002; Frederickson et al., 2003; Seymour et al., 2006; Lebret et al., 2016) and no common protocol exists for sampling and analyzing microbial communities (Zinger et al., 2012; Lebret et al., 2016).

Despite all these efforts, there is still a debate regarding the dispersal limits of microorganisms: is it true that “everything is everywhere, but the environment selects,” as first stated by Beijerinck (1913) and subsequently by Becking (1934). Recent studies emphasize the importance of the environment, stating that microbial diversity is structured by both geography and the environment (Williamson et al., 2008; de Vargas et al., 2015; Malviya et al., 2015), with the presence of microbial spatial patterns (Green and Bohannan, 2006). However, physical barriers in the marine environment are less perceptible than inland barriers, but are still important (Palumbi, 1992, 1994). The Antarctic Polar Front (APF) (Ikeda et al., 1989; Belkin and Gordon, 1996) for instance, can form an open ocean dispersal barrier due to intense currents and a 3–4°C horizontal thermocline (Eastman, 1993; Thornhill et al., 2008). The Southern Indian Ocean is characterized by upper warm and salty water that moves into the South Atlantic Ocean through a system of leakages (Donners and Drijfhout, 2004; Beal et al., 2011), which also regulate the thermohaline circulation cell (Gordon, 1986) impacting the climate globally (Beal et al., 2011). In contrast, the Southern Ocean, of which the flow is dominated by the eastwardflowing Antarctic Circumpolar Current (ACC), is a high nutrient and low chlorophyll region with evidence of iron limitation, characterized by low phytoplankton biomass, which remains constant throughout the year (Popova et al., 2000). The presence of fronts such as the APF has been shown to influence the genetic flow for larger eukaryotes such as worms (Thornhill et al., 2008), toothfish (Shaw et al., 2004), and the brittle star (Hunter and Halanych, 2008). Prokaryote communities also appear to be separated by ocean fronts (Giebel et al., 2009; Wilkins et al., 2013; Baltar et al., 2016; Milici et al., 2017). Similarly, others have shown that the spatial distribution of phytoplankton groups, identified by pigment data, was highly correlated with ocean surface thermal gradients across the ACC (Mendes et al., 2015). Nevertheless, this study represents the first attempt to study the whole microbial community in one go, which includes viruses and protists.

Here, we present the microbiome (viruses, bacteria, and protists) of two oceanic regions of the Indian Ocean basin (South-West and South-East), and an adjacent oceanic region of the Southern Ocean, which is separated from the Indian Ocean by the APF. Amplicon sequencing was used to identify the microbiota present: the V4 region along the 16S rRNA gene and the V9 region of the 18S rRNA gene were used to analyze the prokaryotic and eukaryotic communities, respectively. Furthermore, the viral fraction together with the dissolved or environmental DNA (eDNA) was analyzed using the metagenome shotgun Illumina-sequencing approach. The microbial eDNA (meDNA) represents the theoretical free meDNA that has been released into the environment (i.e., in our case seawater) without isolating it directly from a target microorganism (Flaviani et al., 2017). As we did not include a nuclease step before extracting the nucleic acids from the virions, our “viral size fraction” is therefore composed of viruses plus a mixture of DNA derived from larger cellular debris or released DNA from microbiota or larger biota living in that environment (Taberlet et al., 2012). Originally, eDNA has been used to determine whether an invasion has taken place (Dejean et al., 2012) or to track an endangered species (Ikeda et al., 2016). It has been proposed that eDNA could be used as a an effective monitoring tool without the need to observe an organism *in situ* (Valentini et al., 2016); however, the eDNA concept does not include the microbial community in its definition of cellular organism (i.e., anything that can pass through a 0.5 mm mesh). Here, we utilize the experimental design presented previously to determine whether the microbiota can be monitored by using the eDNA approach, and if consequently the meDNA (Flaviani et al., 2017) works as a good proxy for describing the microbes present in this body of water.

Despite global efforts to study the microbiome, the majority of studies do not address these communities as a whole with the majority of studies only investigated a single group within the microbial world, only 11.2% monitoring two microbial groups simultaneously and 2.2% looking at the interactions between prokaryotes, eukaryotes, and viruses (Zinger et al., 2011). Throughout this study, an alternative and innovative approach is proposed to study microbial diversity in all its complexity, allowing the detection of the most abundant phylotypes.

## Materials and Methods

### Sampling

**FIGURE 1:**
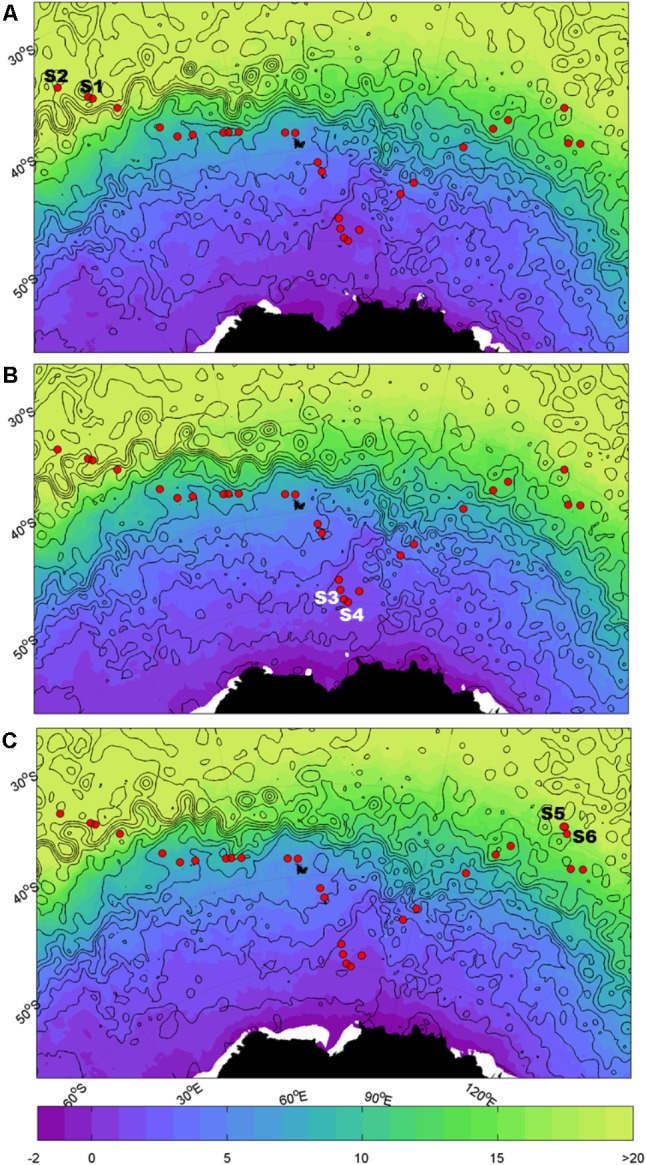
Map of sampling sites. Sample locations for the southern hemisphere overlaid on sea surface temperature (SST, °C; shaded) and dynamic height (m; black contours in 0.2 m intervals from -1.4 m to 1.4 m). Closed contours indicate eddies. The Agulhas Return Current (ARC) is indicated by the band of tightly spaced contours at ~40°S; south of the ARC is the Antarctic Circumpolar Current (ACC). **(A)** Refers to 15/02/12, **(B)** refers to 29/02/2012, and **(C)** refers to 14/03/2012.

Samples were collected during the Great Southern Coccolithophore Belt expedition (GSCB-cruise RR1202) (Balch et al., 2016) at six stations from three oceanic regions (**Table 1**). Given the influence of the Agulhas Return Current in the region off Africa, daily maps of absolute dynamic topography, and sea surface temperature were used to examine the mesoscale circulation of the southern hemisphere oceanic regions in 6 months prior to sampling at the station. Images for **Figure 1** were selected from the 2-month period prior to sampling at intervals of 2 weeks. The absolute dynamic topography fields were calculated by Aviso at 1/4 degree horizontal resolution from all remotely sensed altimetry mission data available at a given time referenced to a 20-year mean (Rio et al., 2013). High resolution (1/20 degree) sea surface temperature data were produced from the Operational Sea Surface Temperature and Ice Analysis (OSTIA) system using both *in situ* and satellite data (Donlon et al., 2012). Stations S1 and S2 were located in the South-West Indian Ocean, stations S3 and S4 in the Southern Ocean, and stations S5 and S6 in the South-East Indian Ocean (**Figure 1**). The locations of the sampling stations along the transect from the south Indian Ocean to the Southern Ocean were mapped using M_map toolbox for Matlab (**Figure 1**).

**Table 1:** Sampling location information.

Station	Location	Date of collection (dd/mm/yy)	Latitude	Longitude	Depth (m)	Temperature^∗^ (°C)
S1	SW Indian Ocean	20/02/12	−38,315	40,958	5	20.83
S2		22/02/12	−35.507	37.458	49.089	19.98
S3	Southern Ocean	06/03/12	−57.598	76.508	41.855	1.38
S4		06/03/12	−58.71	76.89	40.93	1.24
S5	SE Indian Ocean	17/03/12	−39.475	108.935	44.978	16.23
S6		19/03/12	−42.082	113.400	60.55	12.95

*GPS coordinates are provided in decimal degrees. Sampling depth refers to the depth of the chlorophyll-a maximum. ^∗^Converted using the International Temperature Scale of 1990.*

At each station, 1 l of seawater from the chlorophyll maximum layer was sampled by a conductivity temperature depth (CTD) rosette sampler on-board the R/V Roger Revelle. An aliquot of 250 ml was filtered through a 0.45-μm polycarbonate filter. DNA extraction of material retained on the filter was performed using the Qiagen DNeasy Blood and Tissue protocol (QIAGEN, Valencia, CA, United States). The DNA was stored at −21°C and subsequently transferred to Plymouth, United Kingdom, for further processing. An additional 50 ml sample of filtered water was stored at 4°C in the dark for further processing in the laboratory after the cruise.

### Preparation and Sequencing of the >0.45 μm Fraction

For the prokaryotic community composition analysis, the V4 region of 16S ribosomal RNA gene was amplified using the universal primer pair 515F/806R and Illumina tagged primers (Caporaso et al., 2012). Eukaryotes were characterized using the 18S ribosomal RNA gene, using primer pair 1391F/EukB, and Illumina tagging to amplify the V9 region (Stoeck et al., 2010). First, a real-time PCR was run for each sample to determine the mid-exponential threshold of each reaction. For all PCRs, 1–5 μl of DNA, corresponding to 1.47–38.52 ng/μl, respectively, were added to 5× Colorless GoTaq Flexi Buffer (Promega, Madison, WI, United States), 1.5 μl MgCl_2_ Solution 25 mM (Promega, Madison, WI, United States), 2.5 μl dNTPs (10 mM final concentration, Promega, Madison, WI, United States), 1 μl Evagreen Dye 20× (Biotium, Fremont, CA, United States), 0.1 μl GoTaq DNA Polymerase (5 U/μl – Promega, Madison, WI, United States), and sterile water was added to reach the final volume of 25 μl for each reaction. The PCRs were run on a Corbette Rotor-Gene 6000 (QIAGEN, Valencia, CA, United States), with initial denaturation at 94°C for 3 min, followed by 40 cycles of a three step PCR: 94°C for 45 s, 50°C for 60 s, and 72°C for 90 s. Fluorescence in the green channel was recorded at the end of each annealing/extension step. The cycle threshold of the amplification in the exponential phase was recorded for each sample.

The triplicate designed was performed. Briefly, each real time PCR was carried out in triplicate on a unique aliquot of DNA subsampled from the same extraction, and sequenced using single end reads. Through comparing PCR replicates, it is possible to identify overall differences (e.g., we can identify if one of the PCRs is significantly different to others with respect to the number of common/unique OTUs) and also get a sense of which level of diversity can be captured with confidence. Specifically, we can report that OTUs are likely to be observed across all PCRs and in what abundance. Second, a standard PCR amplification was carried out in triplicate and run with the same conditions as the first real-time PCR, excluding the addition of the Evagreen Dye, until the previously determined cycle threshold was reached. PCR products were then run on a 1.4% agarose gel to confirm the success of the amplification and the product size of the amplification. The bands were cut from the gel and purified using the Zymoclean Gel DNA Recovery Kit (Zymo Research, Irvine, CA, United States). Quantity and quality were verified with a NanoDrop 1000 (Thermo Fisher Scientific, Wilmington, DE, United States) and QuantiFluor E6090 (Promega, Madison, WI, United States). Since the V4-16S and V9-18S amplicons were amplified using their replicate specific Illumina tagged primers (Caporaso et al., 2012), the PCR products were combined in equimolar concentrations as measured on the Bioanalyzer (Agilent Technologies, Cheshire, United Kingdom). The final pooled samples were denatured and diluted to 6 pM and mixed with 1 pM PhiX control (Illumina, San Diego, CA, United States), read 1 sequencing primer was diluted in HT1, before the flowcell was clustered on the cBOT (Illumina, San Diego, CA, United States). Multiplexing sequencing primers and read 2 sequencing primers were mixed with Illumina HP8 and HP7 sequencing primers, respectively. The flowcell was sequenced (100 pair end-PE) on HiSeq 2500 using SBS reagents v3.

### DNA Extraction, Preparation, and Sequencing of the <0.45 μm Fraction

The whole 50 ml filtrate (hereafter mention as permeate, without the inclusion of a nuclease pre-treatment) was subjected to a nucleic acid extraction procedure (one sample per station). To the filtered seawater, we added 100 μl of proteinase K (10 mg/ml; Sigma-Aldrich, St Louis, MO, United States) and 200 μl of 10% SDS (Sigma-Aldrich, St Louis, MO, United States). Subsequently we incubated the solution for 2 h at 55°C with constant rotation. The lysate was then collected through multiple centrifugations on a Qiagen DNeasy Blood and Tissue column (QIAGEN, Valencia, CA, United States). The standard Qiagen protocol was followed with 20 μl nuclease-free water (SIGMA, St Louis, MO, United States) used as the elution agent. Quantity and quality were determined using the NanoDrop 1000 (Thermo Fisher Scientific, Wilmington, DE, United States) and QuantiFluor E6090 (Promega, Madison, WI, United States). Two hundred microliters of DNA (<40 ng) was fragmented using a Bioruptor [Diagenode, Seraing (Ougrée), Belgium] on medium for 15 bursts of 30 s with a 30-s pause and concentrated to 30 μl on a Minelute column (Qiagen, Valencia, CA, United States). Fragments were made into libraries using the Nextflex ChipSeq library preparation kit (Bioo Scientific, Austin, TX, United States) without size selection and with 18 cycles of PCR amplification. Bioanalyzer (Agilent Technologies, Cheshire, United Kingdom) as part of library enrichment, Netflex adapter sequences are illustrated on Supplementary Figure S1. Analysis indicated that the final library contained inserts between 30 and 870 bp. The library was multiplexed with other samples and sequenced (100 PE) on a HiSeq 2000 using RTA1.9 and CASAVA1.8 (Illumina, San Diego, CA, United States).

The raw sequences are available at the European Nucleotide Archive (ENA) under accession number PRJEB16346 and PRJEB16674.

### Bioinformatics Pipeline for the Prokaryotic and Eukaryotic Barcodes

The bioinformatics pipeline followed is described in Flaviani et al. (2017). The quality of the reads was first assessed using Fast-QC^1^. The FASTX-Toolkit^2^ was used to trim the first and last 10 bases to remove low quality nucleotides, and subsequently to filter out any reads with fewer than 95% of nucleotide positions called with a quality score of 20. Only the forward read (read 1) was used for the analysis, reverse read (read 2) were dropped due to low-quality. Trimmed and cleaned reads (**Table 2**) from each of the triplicate V4-16S and V9-18S PCRs were pooled in order to assign OTUs using Qiime (Caporaso et al., 2010) with 97% similarities for clustering and Swarm analysis (Mahé et al., 2014), respectively. Taxonomy for both 16S and 18S rRNA genes was assigned using BLASTn implemented in Qiime [1.8] (Caporaso et al., 2010) using the database (db) SILVA version 119 (Quast et al., 2013) (hereafter refer to as SILVA) with a minimum *e*-value of 10e–05.

**Table 2:** Stepwise processing of prokaryote (16S) and eukaryote (18S) sequences.

	Sample	(a)	(b)	(c)	(d)	(e)	(f)
16S	S1 Rep1	1,331,542	773,343	741,033	20,381	705,707	10,281
	S1 Rep2	1,695,911	1,161,634	1,117,576	30,642	1,072,726	15,398
	S1 Rep3	1,626,930	863,867	841,639	24,756	813,380	12,626
	S2 Rep1	983,760	443,622	437,790	17,141	374,459	6,775
	S2 Rep2	1,458,024	627,698	619,255	25,586	525,969	10,078
	S2 Rep3	1,550,314	646,303	637,701	25,208	543,433	9,683
	S3 Rep1	1,491,664	622,030	609,658	18,305	265,157	6,027
	S3 Rep2	1,409,872	795,524	781,413	19,487	353,430	6,170
	S3 Rep3	1,754,942	878,836	864,910	24,344	502,820	9,382
	S4 Rep1	974,224	438,389	434,686	15,668	276,871	5,349
	S4 Rep2	1,609,312	721,401	714,793	20,437	422,624	7,078
	S4 Rep3	1,468,624	795,217	788,622	24,338	567,976	9,182
	S5 Rep1	1,497,998	805,139	785,754	27,469	557,937	10,561
	S5 Rep2	838,777	725,672	706,520	27,206	510,315	10,192
	S5 Rep3	1,253,530	725,301	708,433	28,896	518,912	11,215
	S6 Rep1	1,477,596	664,590	659,890	16,141	205,402	5,563
	S6 Rep2	1,695,898	761,187	755,509	18,741	239,579	6,271
	S6 Rep3	771,891	696,673	691,459	17,978	240,741	6,058
Total 16S				12,896,641	133,550^∗∗^	8,697,438	48,923^∗∗^
18S	S1 Rep1	1,529,536	305,949	223,814	2,972	222,556	1,714
	S1 Rep2	1,614,464	374,041	275,201	3,271	273,710	1,780
	S1 Rep3	1,695,911	419,375	308,208	3,470	306,574	1,836
	S2 Rep1	1,258,768	269,903	179,753	4,499	177,824	2,735
	S2 Rep2	1,626,930	528,707	354,840	5,454	352,118	3,506
	S2 Rep3	1,491,664	425,343	286,080	4,401	283,533	3,260
	S3 Rep1	1,331,542	417,425	33,738	1,043	33,347	288
	S3 Rep2	1,505,002	80,678	7,279	4,369	6,977	163
	S3 Rep3	1,685,214	583,156	71,509	4,595	70,284	1,469
	S4 Rep1	1,392,915	323,407	38,196	4,664	37,698	382
	S4 Rep2	1,393,132	387,326	50,317	6,228	49,754	520
	S4 Rep3	1,403,962	389,608	47,930	5,807	47,464	365
	S5 Rep1	1,188,018	336,163	202,101	880	199,824	2,222
	S5 Rep2	1,799,244	461,542	278,117	1,083	275,184	2,521
	S5 Rep3	1,238,172	310,004	186,059	831	183,800	2,142
	S6 Rep1	838,777	14,341	9,291	679	8,807	559
	S6 Rep2	1,253,530	306,231	195,247	465	193,024	2,146
	S6 Rep3	1,284,848	345,184	223,715	2,694	221,307	2,194
Total 18S				2,971,395	30,169^∗∗^	2,943,785	9,806^∗∗^

*The raw sequence reads (a) were first pre-processed to remove adapters (b), and then trimmed and cleaned (c), before OTUs were assigned (d). Furthermore, singletons were removed (e), and OTUs assigned to this selection (f). The number of unique OTUs was calculated for 16S and 18S-derived datasets. ^∗^For the 16S, dataset chloroplast and mitochondrial sequences were removed at this stage.*

### Bioinformatics Pipeline of the 0.45 μm Permeate

As shown in Flaviani et al. (2017), the pair-end reads (**Table 4**) were assembled into contigs using a De-Bruijin *de novo* assembly program in CLC Genomic Workbench version 7.1.5 (CLCbio, Cambridge, MA, United States) using global alignment with automatic selection of bubble and word size, minimum contig length of 250, mismatch cost of 2, insertion and deletion cost of 3, length fraction of 0.5, and similarity threshold of 0.8. Contigs were annotated using BLASTX (Altschul et al., 1990) against a Virus db (courtesy of Dr Pascal Hingamp), containing Refseq curated viral genomes together with additional new genomes (Mihara et al., 2016). The top hits from all blast searches were selected through the use of a parser Perl script^3^. The ICTV db 2013 v1 implemented with the NCBI taxonomy was utilized to create a viral taxonomy catalog, which was then merged, using R, with the blast output to assign taxonomy.

### Visualization of Community Composition

Krona tools (Ondov et al., 2011) were used to visualize community composition as characterized by the SILVA, Refseq, and Virus db genes taxonomy assignments. Venn diagrams were created using the R package VennDiagram_1.6.17 on R version 3.3.0 (2016-05-03) to determine the number of shared OTUs and phylotypes among sequencing methods used.

### Statistical Analyses

In order to filter the data for singletons, we used the T1 strategy described in Flaviani et al. (2017): i.e., only one read was present for a defined OTU in each replicate before running analyses. Chloroplasts and mitochondria sequences were removed from the prokaryotic dataset prior to the analyses, because they are representatives of the eukaryotic fraction.

Statistical analyses, for both 16S and 18S datasets, were performed under R version 3.3.0 (2016-05-03) combining functionality of the following R packages: reshape 2_1.4.1, reshape_0.8.5, gclus_1.3.1, GGally_1.0.1, scales_0.4.0, car_2.1-2, picante_1.6-2, nlme_3.1-125, ape_3.4, plyr_1.8.2, amap_0.8-14, gridExtra_0.9.1, ggplot2_2.1.0, clusterSim_0.44-2, MASS_7.3-45, cluster_2.0.3, vegan_2.2-1, lattice_0.20-31, permute_0.8-3, sfsmisc_1.1-0. Prior to analyses, the number of reads of prokaryotic and eukaryotic sequences were normalized to the minimum number of reads to avoid bias caused by differences in sequencing depth. Alpha diversity was estimated based on OTU richness. To further analyze the community diversity, analysis of variane (ANOVA) was used to determine if the alpha diversity was statistically different between stations; this analysis was performed using R package *car* using the same two parameters that were utilized in the permutational multivariate (PERMANOVA). Finally, the Tukey’s *post hoc* test based on OTUs observed was performed to test if the number of OTUs varied between locations. Beta diversity was estimated using the *vegan* package based on the Bray–Curtis distance, and plotted as hierarchical clustering. Using the full (not normalized) dataset, we calculated relative abundance for each group and plotted these using the *ggplot2* package. In order to test if community composition was significantly different between sampling stations PERMANOVA analyses were performed using *Adonis* from the vegan package, taking into consideration both temperature and location.

### Statistical Analyses of the <0.45 μm Permeate

Due to the lack of replication of the viral sample, we used Log likelihood ratio statistics to test the goodness of fit for two models. The first model (H0) implied that pairwise sampling stations grouped by location (South-West Indian Ocean and Southern Ocean; South-West Indian Ocean and South-East Indian Ocean; Southern Ocean and South-East Indian Ocean) had the same underlying viral distribution. The second model, the alternative hypothesis (H1) implied that the distribution of viruses depended on the location. We then computed a *p*-value based on the likelihood ratio.

Comparison of prokaryotic and eukaryotic amplicons with the metagenome was run through presence–absence analyses plotted as Venn diagrams using R package VennDiagram_1.6.17. For the metagenome fraction, we used the Refseq annotation while prokaryote and eukaryote taxonomy was assigned using Silva. In order to avoid conflicts in OTU annotation or variation in names in the different dbs, we ran the analysis at genus level (or the first available taxonomic level above).

## Results

Measurements of absolute dynamic topography and sea surface temperature in the months during and prior to the sampling of the southern hemisphere samples indicate that stations S1 and S2 were not directly influenced by the Agulhas Return Current or Antarctic Circumpolar Currents (Rusciano et al., 2012) at the time of sampling (**Figure 1**). This confirms that stations S1 and S2 are representative of the greater south-western Indian Ocean gyre, while stations S3 and S4 are located south of the APF in the Southern Ocean (∼1000 km north of Antarctica) and stations S5 and S6 were north of the ACC representative of the south-eastern Indian Ocean.

### Prokaryotic Diversity and Composition in the >0.45 μm Fraction

A total of 12.9 million prokaryotic sequences, obtained for all six samples, clustered into 133,550 OTUs. When singletons, chloroplast, and mitochondria OTUs were removed, a total of 8.7 million sequences clustered into 48,923 OTUs (**Table 2**). Of these, 44.37% were shared across the six locations and 1.65% shared across all six stations (Supplementary Figure S2a). Specifically 31.17% of the OTUs were unique to the South-West Indian Ocean, 23.30% present exclusively in the Southern Ocean, and 15.04% belonging to the South-East Indian Ocean (**Table 3**).

**Table 3:** Number of OTUs found uniquely at each station and location.

	16S				18S			
	Unique to station	%	Unique to location	%	Unique to station	%	Unique to location	%
S1	7,827	16	15,252	31.17	1,394	14.22	4,308	43.93
S2	4,854	9.92			2,725	27.79		
S3	4,503	9.2	11,403	23.31	98	1	388	3.96
S4	4,111	8.4			221	2.25		
S5	4,486	9.17	7,357	15.04	1,293	13.19	2,578	26.29
S6	1,442	2.95			892	9.1		

The prokaryotic fraction was dominated by known bacterial sequences (average 88.34 ± 0.08%, min = 79.84% in S1, and max = 97.45% in S6) whereas reads with no annotation represented on average 11.01 ± 0.09% of the full dataset (Supplementary Table S1) (min = 0.66% in S6 and max = 22.59% in S3). Archaea were identified in 0.65 ± 0.82% of the sequences (min = 0.01% in S4 and max = 1.89% in S6, Supplementary Table S1). The bacterial fraction was dominated by the phylum Proteobacteria, representing on average 49.55 ± 16.59% of the sequences (min = 24.55% in S2 and max = 64.91% in S4; Supplementary Table S1). This group could be further separated into the γ-Proteobacteria (average = 22.93 ± 11.64%, min = 8.04% in S2, and max = 32.32% in S3), α-Proteobacteria (average = 22.88 ± 8.2%, min = 11.28% in S2, and max = 36.36% in S4) and δ-Proteobacteria (average = 1.39 ± 0.96%, min = 0.24% in S4, and max = 4.11% in S2). Cyanobacteria represented the second most dominant phylum, constituting on average 21.34 ± 23.54% with a minimum of 0.03% in S4 and reaching a maximum of 58.86% in S2 (Supplementary Table S1). The third most represented phylum was Bacteroidetes with an average of 12.76 ± 8.38% (min = 3.26% in S1 and max = 24.37% in S4), mainly due to a high presence of sequences identified as class Flavobacteriia (average = 12.39 ± 8.3%, min = 3.21% in S1, and max = 23.86% in S4).

**FIGURE 2:**
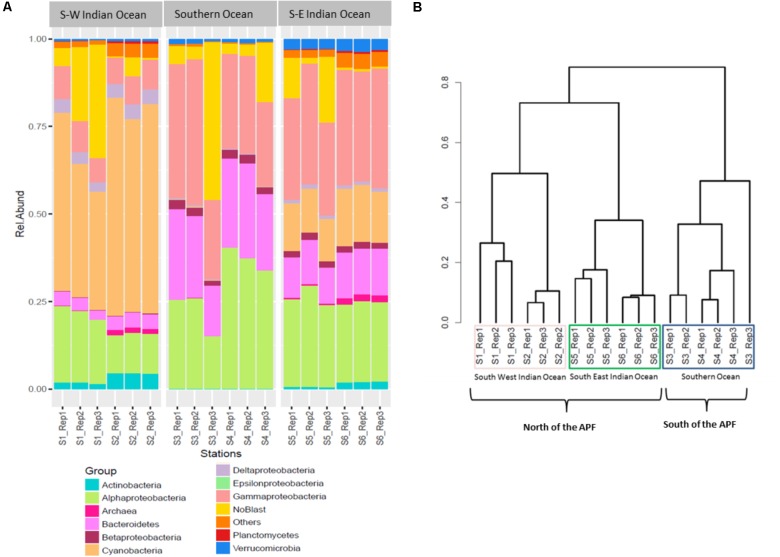
Prokaryote community composition. **(A)** Prokaryotic relative abundance composition on reads; **(B)** Bray–Curtis hierarchical clustering.

The relative abundances of the three most common bacterial groups varied between stations (**Figure 2A**). The South-West Indian Ocean stations S1 and S2 were both dominated by Cyanobacteria, followed by α-Proteobacteria and γ-Proteobacteria. The Southern Ocean station S3 composition was dominated by γ-Proteobacteria, α-Proteobacteria, and Bacteroidetes, while station S4 by α-Proteobacteria, then γ-Proteobacteria and Bacteroidetes. Both stations located in the South-East Indian Ocean (S5 and S6) were dominated by γ-Proteobacteria, α-Proteobacteria, and Cyanobacteria (**Figure 2A**).

### Locations Comparison of Prokaryotic Communities

Bacterial community OTUs composition differed significantly between the three main locations (PERMANOVA *F*_2,12_ = 64.549, *p* = 0.001^∗^). The Bray–Curtis dissimilarity matrix visualized through hierarchical clustering shows that the six stations clustered according to the three locations that correspond to the South-West Indian Ocean, South-East Indian Ocean, or Southern Ocean (**Figure 2B**). OTU richness was also significantly different between locations (ANOVA *F*_2,12_ = 5.28, *p* = 0.0227^∗^). A *post hoc* Tukey’s test showed that only the South-West Indian Ocean and the Southern Ocean were significantly different in the number of OTUs (*p* adj > 0.01).

### Eukaryote Biodiversity and Community Composition in the >0.45 μm Fraction

For the eukaryotic fraction, 5.94 million sequences clustered into 30,169 OTUs. After the removal of singletons, a total of 5.88 million sequences clustered into 9,806 OTUs unique in our dataset (**Table 2**). Of these, 32.46% were shared across the three locations and 1.96% shared across all six stations (Supplementary Figure S2b). Specifically, 43.93% of the eukaryotic OTUs were unique to the South-West Indian Ocean, 3.96% present exclusively in the Southern Ocean and 26.29% belonging to the South-East Indian Ocean (**Table 3**).

The majority of OTUs from the eukaryote fraction corresponded to known sequences with uncharacterized sequences only accounting for 0.32 ± 0.31% (Supplementary Table S2). Eukaryote communities at all stations were dominated by the super-group SAR (Stramenopiles, Alveolata, Rhizaria) representing 85.52 ± 9.80% of all sequences (min = 69.99% in S4 and max = 93.27% in S6; Supplementary Table S2). For this super-group, the major representative was the super phylum Alveolata (average = 83.52 ± 9.80%, min = 68.98% in S4, and max = 91.66% in S1, Supplementary Table S2) followed by Rhizaria (average = 1.72 ± 0.91%, min = 0.41% in S4, and max = 2.84% in S2, Supplementary Table S2). The second main group was represented by the division Haptophyta (average = 9.42 ± 9.80%, min = 1.15% in S2, and max = 27.17% in S4, Supplementary Table S2) with Prymnesiophyceae as the main representative (average = 9.10 ± 11.10%, min = 1.15% in S2, and max = 27.17 in S4, Supplementary Table S2) with the genus *Phaeocystis* representing 26.45% of S4 and 17.71% of S3, while less than 1% at the remaining stations (Supplementary Table S2).

**FIGURE 3:**
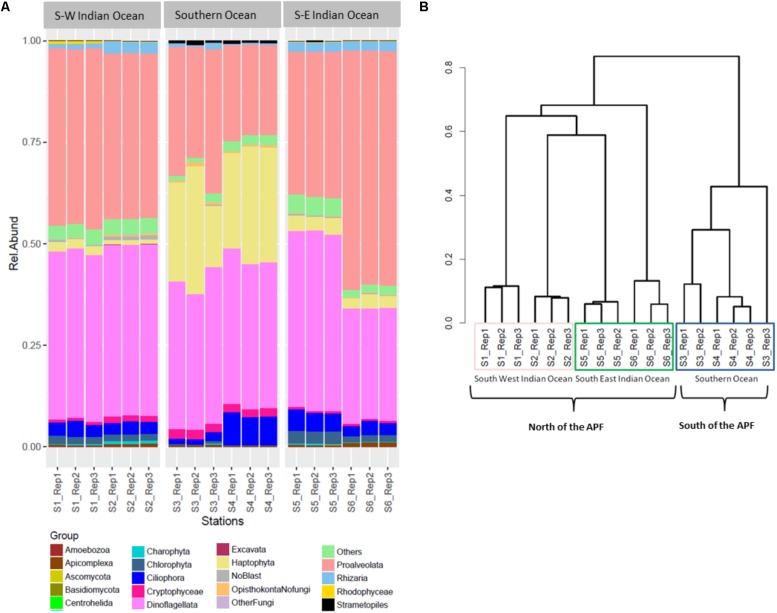
Eukaryote community composition. **(A)** Eukaryote relative abundance composition on reads; **(B)** Bray–Curtis hierarchical clustering.

The three most abundant eukaryotic groups at each station, annotated per station with SILVA level four of taxonomy (L4), were as follows: South-West Indian Ocean station S1 was characterized by Protoalveolata (43.86%), Dinoflagellata (41.35%), and Ciliophora (3.40%); all belonging to the super-group SAR (**Figure 3A**). Station S2 was represented by Dinoflagellata (42.14%), Protoalveolata (40.73%), and Ciliophora (3.12%). Southern Ocean station S3 was dominated by Dinoflagellata (37.57%), Protoalveolata (33.80%), and Haptophyta–Prymnesiophyceae–*Phaeocystis* (18%), while the second polar station S4 was characterized by Dinoflagellata (36.47%), Haptophyta–Prymnesiophyceae–*Phaeocystis* (26%), and Protoalveolata (22.57%). Station S5 in the South-East Indian Ocean was composed of Dinoflagellata (43.78%), Protoalveolata (35.68%), and Ciliophora (4.71%), whereas station S6 was dominated by Protoalveolata (57.73%) followed by Dinoflagellata (27.58%) and Ciliophora (3.21%; **Figure 3A**).

### Locations Comparison of Eukaryotic Community

Eukaryotic community OTUs composition differed between the three main locations (**Figure 3B**; PERMANOVA *F*_2,12_ = 67.38, *p* = 0.001^∗^). Bray–Curtis dissimilarity matrix analyzed through hierarchical clustering shows how the six stations clustered as two different locations separated by the APF. Furthermore, we observed the clustering of station S2 with station S5, suggesting some interchange across the Southern Indian Ocean north of the ACC (**Figure 3B**). OTU richness was significantly different between locations (ANOVA *F*_2,12_ = 30.22, *p* < 0.001^∗^). A *post hoc* Tukey’s test showed that both the South-West and the South-East Indian Ocean were significantly different in the number of OTUs to the Southern Ocean (*p* adj > 0.0001), whilst the two Southern Indian Ocean station were not significantly different (*p* adj = 0.851).

### Biodiversity in the <0.45 μm Filter Permeate: Viral Contigs

The raw reads were assembled into a range between 5 and 35 thousand contigs depending on sampling station (**Table 4**). A selection of contigs, chosen for the presence of key viral features such as the presence of viral tail components, major head protein, and viral capsid proteins, was examined to confirm positive viral identification after annotation with the viral db (Supplementary Figure S3).

**Table 4:** From raw reads to number of contigs assembled using CLC genomic workbench stepwise processing of metagenome.

Dataset	Sample	Raw reads	Reads used	Number of contigs	Average contig length	Smallest contig	Largest contig	N50
Metagenome	S1	90,672,808	10,036,627	4,962	1,045	240	74,442	7,239
	S2	16,569,598	16,569,598	35,358	1,060	206	282,176	6,999
	S3	21,466,152	21,466,152	20,597	1,492	206	388,233	3,668
	S4	21,840,372	21,840,372	15,844	1,492	230	563,674	8,321
	S5	14,268,562	14,268,562	18,540	1,312	217	478,618	5,267
	S6	41,108,086	41,108,086	7,539	2,092	249	1,026,488	161,188

**FIGURE 4:**
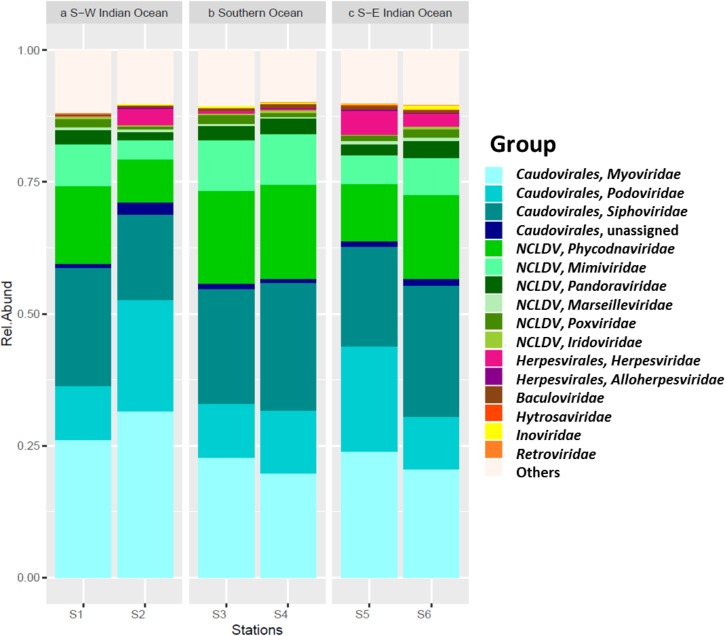
Viral contigs. Relative abundance of main viral group by station, separated per location.

Viral sequences across the south Indian Ocean and the Southern Ocean were dominated by members the order *Caudovirales* with an average of 60.57 ± 5.96% (**Figure 4**; Supplementary Table S3); the lowest abundance for this order was seen at station S3 (55.71%), the maximum was observed at station S2 (71.14%; Supplementary Table S4). On average, members of the family *Myoviridae* represented 24.07 ± 4.30%, *Siphoviridae* 21.39 ± 3.32%, and *Podoviridae* 13.92 ± 5.21% of all the caudoviruses. Myoviruses were the most abundant in four of the six stations, representing on average 39.56 ± 4.00% of this order (min 34.80% S4, max: 44.32% S2), while siphoviruses were the most abundant representatives of the order *Caudovirales* in both stations S4 and S6 (43 and 44%, respectively) (Supplementary Table S3).

**FIGURE 5:**
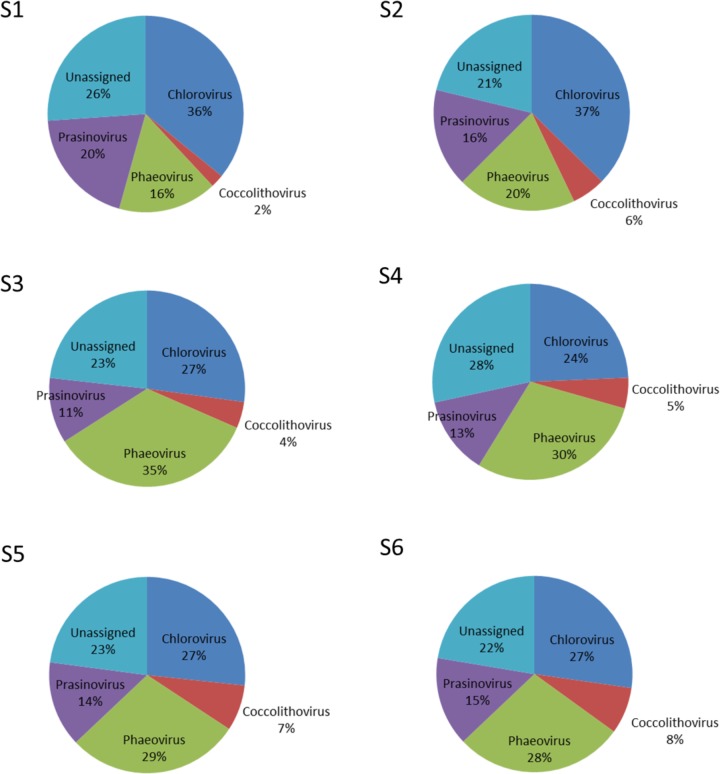
Viruses of the family Phycodnaviridae at the six sampling stations.

Nucleocytoplasmic large DNA viruses (NCLDV) represented the second most dominant viral group comprising 26.35 ± 6.85% of the virus genes annotated (**Figure 4**; Supplementary Table S3), with a minimum at station S2 (15.32%) and a maximum at station S3 (32.67%). Phycodnaviruses represented about half (49.67 ± 2.41%) of the NCLDVs (**Figure 4**) or 13.13 ± 3.64% of all sequences (Supplementary Table S3). This viral family was dominated by generic chloroviruses in the South-West Indian Ocean (S1 and S2), phaeoviruses in the Southern Ocean (S3 and S4), and by both chloroviruses and phaeoviruses in the South-East Indian Ocean (S5 and S6; **Figure 5**). Many of the phycodnaviruses could not be assigned to any specific genus (21 to 28%), while prasinoviruses (11 to 20%) and coccolithoviruses (2 to 8%) made up the remainder across all six stations (**Figure 5**).

The NCLDVs were further characterized by the strong presence of presumptive members of the family *Mimiviridae* (26.86 ± 2.56%, **Figure 4**), comprising on average 7.19 ± 2.34% (min = 3.64% S2, max = 9.56% S3 and S4; Supplementary Table S4). Sequences from the order *Herpesvirales* represented 1.97 ± 1.92% of the viruses, with a minimum (0.14%) in the South-West Indian Ocean S1 and a maximum (4.78%) in the South-East Indian Ocean S5 (Supplementary Table S3).

### Spatial Patterns in Viral Distributions

Due to the way in which we collected our metagenomic samples, and in particular the absence of replication, we used a log likelihood ratio statistic to look at community differences in the three locations. In our model, we started with a null hypothesis that the same underlying viral distribution across all three locations exist and consequently testing the influence of the polar front on viral dispersal. Results from this analysis show that viral community composition south of the APF were significantly different from stations located north of the front (pchisq = 8.89E-120; **Table 4**). We also tested if the three locations had the same underlying viral distribution; resulting in the three areas being significantly different to each other (pchisq = 2.68ˆE-60 to 7.32E-131; **Table 5**).

**Table 5:** Pairwise log likelihood ratio statistics to test for differences in viral community composition between the three ocean regions sampled in this study.

	SWID:SO	SWIO:SEIO	SO:SEIO	Above PF:Below PF
null.loglike	96484.7	97847.73	56698.81	125432.7
alternative.loglike	96787.33	97901.93	56838.58	125709.7
Lrs	605.2532	108.3967	108.3967	554.1192
p chisq	7.32E-131	2.43E-23	2.68E-60	8.89E-120

*Analyses were performed at the level of Order (Caudovirales, Megavirales, Herpesvirales, Other). SWIO, South-West Indian Ocean; SO, Southern Ocean; SEIO, South-East Indian Ocean; PF, Polar Front.*

### Comparison of the Compositions of Permeate vs. Cellular Fraction

**FIGURE 6:**
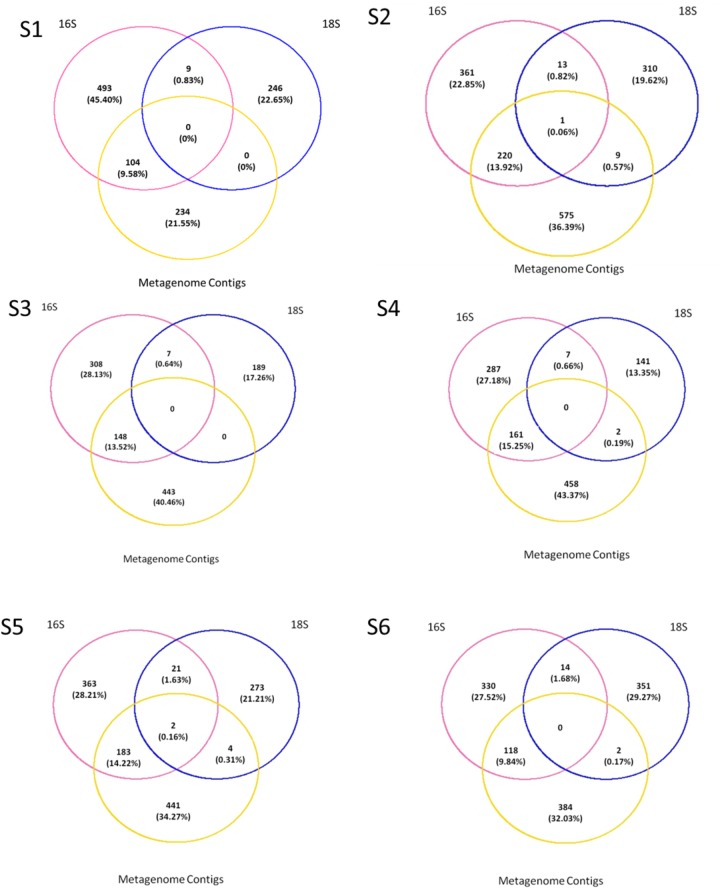
Presence–absence between the prokaryotes, eukaryotes, and permeate. Comparisons were made on genus as the lowest level available, T1 for the prokaryotes and eukaryotes, and T0 on all contigs blasted with Refseq db.

Presence/absence analyses were performed across all fractions for each station to understand if the metagenome contained unique OTUs that can be due to the presence of meDNA. To do so we compared the “genus” level assignments from the ORFs on metagenomic assembled contigs to the annotations from the amplicon sequences. The majority of the sequences were not shared across the datasets (**Figure 6**). A maximum of 9 out of a possible 320 (0.57% of the overall dataset) eukaryotic genera could be detected in the permeate or meDNA fraction at one station (S2), while two stations (S1 and S3) shared uncommon sequences. A range of bacterial genera (9.58 to 15.25%) could however be found in common between the prokaryotic and meDNA fractions (average 12.77 ± 2.46%). Commonalities between the prokaryotic and eukaryotic db were due to the presence of chloroplast and mitochondria OTUs included in both datasets (**Figure 6**). These were maintained at this stage of the analyses in order to verify possible overlaps between the prokaryotic and eukaryotic datasets.

The five most abundant genera identified within the meDNA permeate at all six stations, included almost half of the phylotypes that were shared between the viral and prokaryote fractions (average = 52.57 ± 9.37%, min = 41% in S2, and max = 63% in S3; **Table 4**). Members of the genera *Alcanivorax* and *Marinobacter* were found at three of the stations (S2, S5, and S6; **Table 6**).

**Table 6:** The five most abundant genera (percentage shown in brackets) found in the <0.45 μm fraction (eDNA) based on contigs annotation using the Refseq database.

S1	S2	S3	S4	S5	S6
***Microbacterium* (33%)**	*Halomonas* (12%)	*Alteromonas* (32%)	*Roseobacter* (18%)	*Alteromonas* (15%)	*Alcanivorax* (16%)
*Erythrobacter* (24%)	*Erythrobacter* (10%)	*Sulfitobacter* (10%)	*Sulfitobacter* (12%)	*Marinobacter* (11%)	***Oceanicola* (12%)**
***Citomicrobium* (4%)**	*Alcanivorax* (7%)	*Halomonas* (8%)	*Thalassolituus* (7%)	***Oceanicola* (8%)**	*Methylophaga* (9%)
*Novosphingobium* (2%)	*Marinobacter* (6%)	***Oceanibulbus* (8%)**	***Hypnomonas* (5%)**	*Thalassolituus* (7%)	*Marinobacter* (9%)
*Arthrobacter* (1%)	*Methylophaga* (6%)	*Erythrobacter* (5%)	*Ruegeria* (4%)	*Alcanivorax* (7%)	***Hyphomonas* (8%)**

*Genera highlighted in bold font were absent from the prokaryotic dataset.*

## Discussion

### Everything Is Everywhere, But the Environment Selects

The comparative analyses of amplicon (prokaryotes and eukaryotes) and metagenomics (viral, eDNA) fractions of the six seawater samples sequenced in this study showed clear patterns in the spatial distribution of microbes, with significant differences of phylotype compositions between the Indian Ocean and the Southern Ocean systems as well as across the three regions that were sampled. We found that the majority of the genomic sequences were only present at a specific station or location, which reinforces the hypothesis that “the environment selects” (Green and Bohannan, 2006). If “everything” were indeed “everywhere,” one would expect that the majority of sequences would be shared between all sampling stations, but this was only the case for 30% of eukaryotes and 44% of prokaryotes. Our study therefore supports other recent evidence that marine microbial communities exhibit distinctive spatial distribution patterns with microbial diversity structured by both geography and environment (Green and Bohannan, 2006; Williamson et al., 2008; de Vargas et al., 2015).

Stations in the Indian and Southern Oceans showed clear differences, with the samples collected at opposite sides of the Indian Ocean basin sharing more taxa than samples at a position that was geographically located between them. Previous studies have shown differences in the prokaryotic community on both sides of the APF (Giebel et al., 2009; Wilkins et al., 2013; Baltar et al., 2016; Milici et al., 2017); here we show that this pattern was especially prominent for eukaryotes, but was also evident for viral communities. We hypothesize that these differences are attributed to physiological limitations that render certain microbial groups unsuited for the conditions other than those they are adapted to. Variations in temperature, nutrients and minerals are quite different between the South Indian and Southern Oceans (Popova et al., 2000; Donners and Drijfhout, 2004; Beal et al., 2011), and it has been previously demonstrated that abiotic factors have a limited effect on community structure (Lima-Mendez et al., 2015).

### Comparison With Previous Studies of Microbial Diversity

The Tara Ocean Expedition has contributed significantly to our understanding of the diversity of microbes in the oceans; reporting, for example, Dinophyceae dominance as OTU richness in the global pico-nanoplankton community, with almost 25,000 of the 87,000 annotated OTUs (28%) for the full eukaryotic dataset being present in more than 40 of the 47 stations (de Vargas et al., 2015). In contrast, we did not see dominance of the class Dinophyceae but a similar ratio of protoalveolates and dinoflagellates. Specifically, Protoalveolata dominated station S6 while Dinoflagellata had higher relative abundances in stations S5 and S4; similar ratios of these microbes were found in stations S1, S2, and S3. Similar results from the Tara Ocean Expedition, the Protoalveolata fraction was dominated by the Syndiniales groups I and II, which were identified with previous nomenclature of MALV-I and MALV-II (de Vargas et al., 2015; Horiguchi, 2015). The two Southern Ocean stations (S3 and S4, sampled at the end of summer, March 2012) had higher abundance of haptophytes, specifically due to presence of *Phaeocystis*. This relates to previous studies on the Southern Ocean, in which diatoms and haptophytes such as *Phaeocystis* were found more abundant in the more nutrient rich polar front regions and continental shelves (Constable et al., 2014); furthermore in the Ross sea *Phaeocystis* was the dominant primary producer for the deeply mixed waters (20–50 m) whereas diatoms dominated highly stratified waters (5–20 m; Arrigo et al., 1999). Specifically *Tara*’s station 85 (sampled during summer, January 2011), based on the Southern Ocean, had higher presence of haptophytes (de Vargas et al., 2015).

The prokaryotic dataset showed dominance of cyanobacteria in the seawater we sampled from South-West Indian Ocean, while the South-East Indian Ocean and Southern Ocean were dominated by proteobacteria. Specifically γ-Proteobacteria dominated both S5 and S6 from the South-East Indian Ocean and S3 from the Southern Ocean, while S4 was dominated by α-Proteobacteria. These results are compatible with what was found during Tara’s sampling expedition where Proteobacteria, specifically α-Proteobacteria, dominated both surface waters and the deep chlorophyll maximum; Cyanobacteria and γ-Proteobacteria were the second most represented groups depending on location (Sunagawa et al., 2015). Similar results were obtained during the ICoMM campaign in the surface open ocean with α-Proteobacteria, γ-Proteobacteria, Cyanobacteria, and Flavobacteria identified as the most abundant groups in the full datasets (Zinger et al., 2011).

As found in the Tara Global Ocean Expedition study (Sunagawa et al., 2015) we confirm that water temperature, which is a major defining characteristic of the different stations north and south of the APF, plays an important role in determining microbial community dispersal. During the International Census of Marine Microbes (ICoMM) 9.5 million DNA prokaryotic sequences clustered into around 120,000 OTUs (Zinger et al., 2011). Our limited dataset (prior to the removal of singletons, chloroplasts and mitochondria OTUs) comprises 12.9 million prokaryotic sequences that clustered into 133,500 OTUs; an increase of 13,500 new OTUs. As seen in the ICoMM dataset (Zinger et al., 2011), when the singletons were removed, chloroplast and mitochondria sequences made up almost half of the OTUs. Nevertheless, different from the ICoMM study, post singleton removal ∼70% of the sequences were retained for both the prokaryotes and eukaryotes, showing that we were able to recover the most abundant phylotypes.

### The Virome

Given the dominance of bacteria in our oceans, the assumption is that most marine viruses are bacteriophages (Wommack and Colwell, 2000). Viruses generally dominate oceanic waters with approximately 10 million viruses per milliliter of seawater (Bergh et al., 1989; Wilhelm and Suttle, 1999; Suttle, 2005; Breitbart, 2012). Metagenomic studies have found that tailed viruses in the order *Caudovirales* are the most abundant in the marine environment (Williamson et al., 2008, 2012; Hurwitz and Sullivan, 2013; Chown et al., 2015) and that generally myoviruses predominate, followed by podoviruses and then siphoviruses. However, it was reported that a hypersaline lagoon was dominated by siphoviruses followed by podoviruses and then myoviruses (Williamson et al., 2008), showing that variation of this group might depend on abiotic conditions that affect the presence of its hosts. This is in agreement with our study where the annotated viral fraction was dominated by viruses in the order *Caudovirales* across all stations with myoviruses being most represented in stations S1, S2, and S5 and siphoviruses in stations S4 and S6. Finally, a similar ratio between myo- and siphoviruses was observed at station S3.

NCLDVs infecting marine protists (Claverie and Abergel, 2013; Blanc-Mathieu and Ogata, 2016), were the second main viral group identified in the permeate, with phycodnaviruses representing almost half of this group in all six samples. This was also previously reported during the Tara expedition, where just over half of the NCLDVs sequences were identified as phycodnaviruses with the other half identified as mimiviruses (Hingamp et al., 2013). The latter was, however, not observed in our data. We saw a higher abundance of presumptive mimiviruses in the Southern Ocean samples (S3, S4), which could be related also to presence of Stramenopiles in these stations – an association seen previously (Hingamp et al., 2013). Furthermore, NCLDVs were the second most abundant group at all our stations, reinforcing the hypothesis that mimiviruses are probably infecting a wide variety of organisms (Claverie et al., 2009). For the family *Phycodnaviridae* we were expecting, from previous studies, the dominance of prasinoviruses (Hingamp et al., 2013). However, we identified the dominance of chloroviruses in stations S1 and S2, phaeoviruses in stations S5 and S6, and an equal ratio of chloro*-* and phaeoviruses in stations S3 and S4.

Chloroviruses are known to infect and replicate in unicellular, chlorella-like green algae collected in freshwater (Van Etten, 2003; Dunigan et al., 2006; Yamada et al., 2006). They have also been reported to be able to replicate in human and mice (Yolken et al., 2014). Presence of chloroviruses in these marine samples suggests that alternative marine eukaryotic hosts exist for these viruses. This is plausible, as our knowledge of viruses infecting marine eukaryotes is still limited to only a few studies (Hingamp et al., 2013), and biases in the isolation procedures against giant viruses are still common place (Van Etten, 2011). High abundance of dinoflagellates in the eukaryotic dataset allows us to hypothesize that these viruses could infect dinoflagellate as alternative hosts; hopefully this can be addressed in future studies. Similarly, phaeoviruses are known to infect a broad range of brown macroalgae (Cock et al., 2010; Schroeder, 2015), so the presence of this group of viruses in absence of their known hosts in the eukaryotic fraction could also indicate an alternative host for this group as well.

### meDNA From 50 ml Is Not a Proxy for Marine Biodiversity Assessments

Presence/absence analyses between the permeate and the cellular fractions collected on the filter showed that on average 13% of the genera were identified in both the prokaryotic and the permeate datasets. The eukaryotic fraction on the other hand could not be described at all (0–0.57%). Four of the five most common prokaryotic genera identified in the permeate, representing almost half of the permeate metagenomic dataset, could be found in the cellular amplicon dataset. This could be indicative of the presence of meDNA from a small proportion of the bacterial community. Interestingly, in three of the four Southern Indian Ocean stations we identified the presence of *Alcanivorax* and *Marinobacter*. These organisms are known to degrade hydrocarbons (Yakimov et al., 1998; Duran, 2010; Moxley and Schmidt, 2012) and could be a sign of oil-containing seawater due to active shipping routes.

The remaining genera, which were not identified on filters but present in the permeate, could represent the presence of small bacteria passing through the 0.45 μm filter (Anderson and Heffernan, 1965; Tabor et al., 1981; Hasegawa et al., 2003; Hahn, 2004), vesicles (Biller et al., 2016) or “bacterial detritus” (Falkowski et al., 2008). We also cannot exclude the hypothesis that some of the “cellular” sequences could be of viral origin, since viral genes have been reported to match genes commonly found in the genomes of their prokaryotic and eukaryotic hosts (Wilson et al., 2005; Baumann et al., 2007; Filée et al., 2007).

## Conclusion

In this study, we showed that prokaryotic, eukaryotic, and viral communities differ in composition in the South Indian Ocean and the Southern Ocean, differences that can be related to open-ocean dispersal barrier such as the APF. Differences in community composition were observed also on the South-West and South-East of the Indian Ocean, with the prokaryotic community being more separated than the eukaryotes, differences that can be attributed to the location of the South-East site being below the Subtropical front (Balch et al., 2016). Differences in the host fraction were reflected into the viral composition across the three sampling location. Furthermore, the increase in haptophytes in the Southern Ocean was reflective of an increase of large eukaryotic viruses. These differences, affecting the microbial communities, can be attributed to the collection location of the South-East samples being below the Subtropical front (Balch et al., 2016). As found in one of the Tara study (Sunagawa et al., 2015), water temperature appear to be a major defining characteristic of the different stations above and below the APF, and played an important role for determining microbial community structure.

This study therefore reinforces the paradigm that “everything is everywhere, but the environment selects” not only for the prokaryotic but also the protists and viral communities, showing that clear differences exist in the spatial distribution of microbial communities due to environmental selection and adaptation. Finally, our results unequivocally demonstrate that the composition of the cellular amplicon fraction differs dramatically from the eDNA or meDNA permeate; therefore, raising the efficacy of using meDNA to monitor aquatic microbial diversity.

This method can be easily implemented in time series monitoring of the marine environment, opening the door to a more integrated approach of oceanographic sampling, thereby allowing for better parameterization of global biological models. In the past, the inability to characterize microbial assemblages through visual identification has created a drawback in marine monitoring (Goodwin et al., 2016). The techniques and methodologies utilized throughout this study provide an alternative cost effective approach to ecosystem integrated monitoring. The identification of dominant and likely the most active marine microbial community through the genetic characterization of smaller volumes of water will hopefully allow for a better integration of microbial data in ecosystem models.

## Author Contributions

FF and DS wrote the manuscript. DS conceived the study. CB and AH collected the samples and prepared the amplicons for Illumina sequencing. FF and CB prepared the sample for metagenome Illumina sequencing. KM and KP prepared the amplicons and metagenome for Illumina sequencing and performed the sequencing. FF, KL, and JS performed bioinformatics and statistical analyses. DS, MP, and ER funded the project. ST prepared and analysed the remotely sensed oceanographic data. All authors reviewed the manuscript.

## Conflict of Interest Statement

The authors declare that the research was conducted in the absence of any commercial or financial relationships that could be construed as a potential conflict of interest.

## Footnotes

1 http://www.bioinformatics.babraham.ac.uk/projects/fastqc/

2 http://hannonlab.cshl.edu/fastx_toolkit/

3 http://www.bioinformatics-made-simple.com
