# Supplementary material for: Distinct Oceanic Microbiomes From Viruses to Protists Located Near the Antarctic Circumpolar Current
Source: Front Microbiol. 2018 Jul 17;9:1474. doi: 10.3389/fmicb.2018.01474 (PMC6056678; doi:10.3389/fmicb.2018.01474)
Supplement: Supplementary file 1 [file Presentation_1.PDF]

Supplementary Material

The Antarctic Polar Front forms a distinct microbial ocean boundary between the Indian and Southern oceans

Flavia Flaviani 1,2, Declan C. Schroeder 2,3,4*, Karen Lebret 2,5, Cecilia Balestreri 2, Joanna L. Schroeder 2, Karen Moore 6, Konrad Pasckiewicz 6, Maya C. Pfaff 7, Edward P. Rybicki 1* *** Correspondence:**

Corresponding Author: dsch@mba.ac.uk

1 Supplementary Table

Supplementary table 1: Prokaryote community composition across stations. Percentage of T1 average of the three replicates. Values are shown only if > 0.5%.

L1	L2	L3	S1	S2	S3	S4	S5	S6	Average
**Archaea**	**Euryarchaeota**	Thermoplasmata						1.71%	
	**Thaumarchaeota**	Marine Group I		0.99%					
	Total Archea			1.45%				1.89%	0.65%
**Bacteria**	**Cyanobacteria**	Cyanobacteria	40.26%	58.79%			12.79%	15.73%	
		Total Cyanobacteria	40.32%	58.86%			12.81%	15.77%	21.34%
	**Proteobacteria**	α-Proteobacteria	20.22%	11.28%	20.94%	36.36%	25.82%	22.69%	
		β-Proteobacteria			2.02%	2.28%	1.91%	1.80%	
		δ-Proteobacteria	4.11%	4.11%			0.99%	1.04%	
		Elev-16S-509	0.89%	0.69%			0.67%	0.83%	
		γ-Proteobacteria	25.50%	8.04%	32.32%	26%	29.99%	32.81%	
		Total Proteobacteria	32.95%	24.55%	55.67%	64.91%	59.40%	59.79%	49.55%
	**Bacteroidetes**	Flavobacteriia	3.21%	3.72%	19.56%	23.86%	11.10%	12.89%	
		Total Bacteroidetes	3.36%	4.17%	20.01%	24.37%	11.44%	13.17%	12.76%
	**Actinobacteria**	Acidomicrobiia	1.72%	4.52%			0.57%	1.99%	
		Total Actinobacteria	1.73%	4.54%			0.60%	2.05%	1.52%
	**Chloroflexi**			0.73%				0.59%	
	**Deferribacteres**			1.36%			0.98%	1.94%	
	**Planctomycetes**			0.67%				0.55%	
	**Verrucomicrobia**	Opitutae			0.95%	0.87%	2.44%	3.07%	
		Total Verrucomicrobia	0.50%	0.58%	0.96%	0.88%	2.87%	3.39%	1.53%
	Total Bacteria		79.54%	96.23%	77.40%	90.67%	88.75%	97.45%	88.34%
**No blast hit**			20.34%	2.31%	22.59%	9.32%	10.82%	0.66%	11.01%

Supplementary table 2: Eukaryote community composition across stations. Percentage of T1 average of the three replicates. Values are shown only if > 0.5%.

L1	L2	L3	L4	L5	S1	S2	S3	S4	S5	S6	Average
**Eukaryota**	**Archaeplastida**	Chloroplastida			2.09%	2.33%	0.70%		3.40%	1.83%	
**Eukaryota**	**Archaeplastida**	Rhodophyceae			0.58%						
**Eukaryota**	**Archaeplastida**	Total Archeaplastida			2.68%	2.42%	0.73%		3.50%	1.94%	2.07
**Eukaryota**	**Cryptophyceae**	Cryptomonadales			0.67%	1.32%	2.15%	2.02%			
**Eukaryota**	**Cryptophyceae**	Total Cryptophyceae			0.72%	1.47%	2.21%	2.02%	0.57%		1.32
**Eukaryota**	**Haptophyta**	Pavlovophyceae			1.09%						
**Eukaryota**	**Haptophyta**	Prymnesiophyceae			0.87%	1.06%	18.82%	27.10%	3.60%	3.18%	
**Eukaryota**	**Haptophyta**	Prymnesiophyceae		*Phaeocystis*	0.05		17.71	26.45	0.96	0.62	
**Eukaryota**	**Haptophyta**	Total Haptophyta			2.33%	1.15%	18.86%	27.17%	3.78%	3.21%	9.42
**Eukaryota**	**Opisthokonta**	Holozoa					0.52%	0.51%			
**Eukaryota**	**SAR**	Alveolata	Dinoflagellata	Dinophyceae	33.54%	31.61%	27.51%	30.40%	31.09%	17.39%	
**Eukaryota**	**SAR**	Alveolata	Dinoflagellata	uncultured eukaryote	4.47%	8.79%	9.16%	5.75%	10.90%	6.65%	
**Eukaryota**	**SAR**	Alveolata	Dinoflagellata	Total Dinoflagellata	41.35%	42.14%	37.57%	36.47%	43.78%	22.58%	37.32
**Eukaryota**	**SAR**	Alveolata	Protoalveolata	Syndiniales	42.37%	40.44%	33.66%	22.55%	35.13%	57.50%	
**Eukaryota**	**SAR**	Alveolata	Protoalveolata	Perkinsidae	1.43%				0.56%		
**Eukaryota**	**SAR**	Alveolata	Protoalveolata	Total	43.86%	40.73%	33.8%	22.57%	35.68%	57.73%	39.06

				Protoalveolata							
			Total Alveolata		91.66%	88.14%	74.79%	68.98%	86.63%	90.93%	83.52
		Rhizaria			1.14%	2.84%	1.34%		2.30%	2.29%	
		Stramenopiles						0.60%			
		Total SAR			92.92%	91.12%	76.61%	69.99%	89.19%	93.27%	85.52
	**uncultured marine eukaryote**				0.77%	1.79%	0.62%		2.08%	0.51%	
	Total Eukaryota				99.72%	99.09%	99.77%	99.99%	99.68%	99.84%	99.68
**No blast hit**						0.91%					

Supplementary table 3: Percentage of contigs annotated using the virus db. Values are shown only if > 0.5%.

Order	Family	S1	S2	S3	S4	S5	S6	Average
Caudovirales	*Myoviridae*	26.03%	31.53%	22.70%	19.73%	23.86%	20.56%	
	Podoviridae	10.33%	21.14%	10.27%	11.89%	20.01%	9.90%	
	Siphoviridae	22.35%	16.16%	21.72%	24.32%	18.93%	24.87%	
	Unassigned	0.71%	2.31%	1.01%	0.77%	1.02%	1.29%	
	Total *Caudovirales*	59.41%	71.14%	55.71%	56.72%	63.82%	56.62%	60.57%
**NCLDVs**	*Ascoviridae*	1.70%	1.28%	1.72%	1.60%	1.35%		
	Iridoviridae	0.57%					0.54%	
	Marseilleviridae	0.57%	0.57%			0.61%	0.65%	
	Mimiviridae	7.92%	3.64%	9.56%	9.56%	5.44%	7.00%	
	Pandoraviridae	2.69%	1.52%	2.77%	3.04%	2.17%	3.23%	
	Phycodnaviridae	13.01%	7.52%	16.28%	16.42%	10.14%	15.39%	
	Poxviridae	1.56%	0.59%	1.59%	0.77%	0.95%	1.51%	
	Total *NCLDV*	28.15%	15.32%	32.67%	32.17%	20.92%	28.85%	26.35%
	Herpesviridae		3.12%			4.55%	2.37%	
	Total *Herpesvirales*		3.42%	0.51%		4.78%	2.58%	1.97%
**Other**	*Baculoviridae*				0.83%	0.66%	0.54%	
	Chlorovirus	1.70%	0.66%	1.35%	1.38%	0.66%	0.65%	
	Inoviridae						0.75%	
	Unassigned	9.62%	8.51%	8.28%	7.46%	8.28%	9.36%	
	Total *Other*	12.31%	10.05%	10.95%	10.45%	10.32%	11.73%	10.97%

2 Supplementary Figures

Full-length indexed PCR product (green indicates library insert)

```
5'-AATGATACGGCGACCACCGAGATCTACACTCTTTCCCTACACGACGCTCTTCCGATCTXXXXXX-//-XXXXXXAGATCGGAAGAGCACACGTCTGAACTCCAGTCACNNNNNNATCTCGTATGCCGTCTTCTGCTTG
   ||||||||||||||||||||||||||||||||||||||||||||||||||||||||||||||||    ||||||||||||||||||||||||||||||||||||||||||||||||||||||||||||||||||||||
   TTACTATGCCGCTGGTGGCTCTAGATGTGAGAAAGGGATGTGCTGCGAGAAGGCTAGAxxxxxx-//-xxxxxxTCTAGCCTTCTCGTGTGCAGACTTGAGGTCAGTGNNNNNNTAGAGCATACGGCAGAAGACGAAC-5'
```

underlining indicates sequences identical to flow cell oligos

Sequencing reads

```
                  Read 1 5'-ACACTCTTTCCCTACACGACGCTCTTCCGATCT-> Index read 5'-GATCGGAAGAGCACACGTCTGAACTCCAGTCAC->
5'-AATGATACGGCGACCACCGAGATCTACACTCTTTCCCTACACGACGCTCTTCCGATCTXXXXXX-//-XXXXXXAGATCGGAAGAGCACACGTCTGAACTCCAGTCACNNNNNNATCTCGTATGCCGTCTTCTGCTTG
   ||||||||||||||||||||||||||||||||||||||||||||||||||||||||||||||||    ||||||||||||||||||||||||||||||||||||||||||||||||||||||||||||||||||||||
   TTACTATGCCGCTGGTGGCTCTAGATGTGAGAAAGGGATGTGCTGCGAGAAGGCTAGAxxxxxx-//-xxxxxxTCTAGCCTTCTCGTGTGCAGACTTGAGGTCAGTGNNNNNNTAGAGCATACGGCAGAAGACGAAC-5'
                                                                          <-TCTAGCCTTCTCGTGTGCAGACTTGAGGTCAGTG-5' Read 2
```

Sequencing Adapter sequences

```
5'-AATGATACGGCGACCACCGAGATCTACACTCTTTCCCTACACGACGCTCTTCCGATCT
5'-AGATCGGAAGAGCACACGTCTGAACTCCAGTCACNNNNNNATCTCGTATGCCGTCTTCTGCTTG
```

R/C of Sequencing adapter sequences

```
5'- GATCGGAAGAGCGTCGTGTAGGGAAAGAGTGTAGATCTCGGTGGTCGCCGTATCATT
5'- CAAGCAGAAGACGGCATACGAGATNNNNNNGTGACTGGAGTTCAGACGTGTGCTCTTCCGATCT
```

Supplementary Figure 1: Nextera adapters sequence

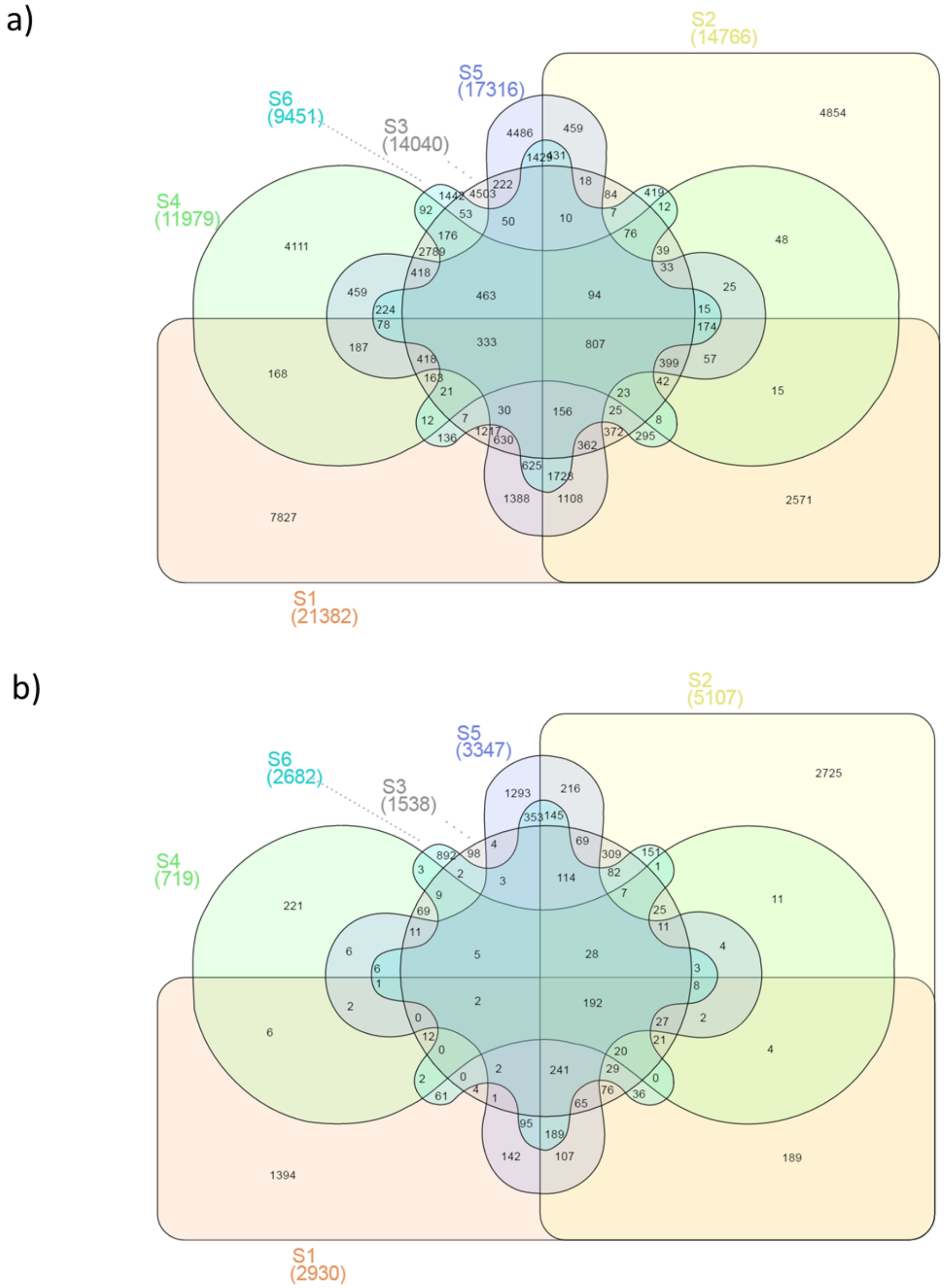

Supplementary Figure 2: Six-way Venn diagram based on OTUs per station. (a) Prokaryotes, (b) eukaryotes.

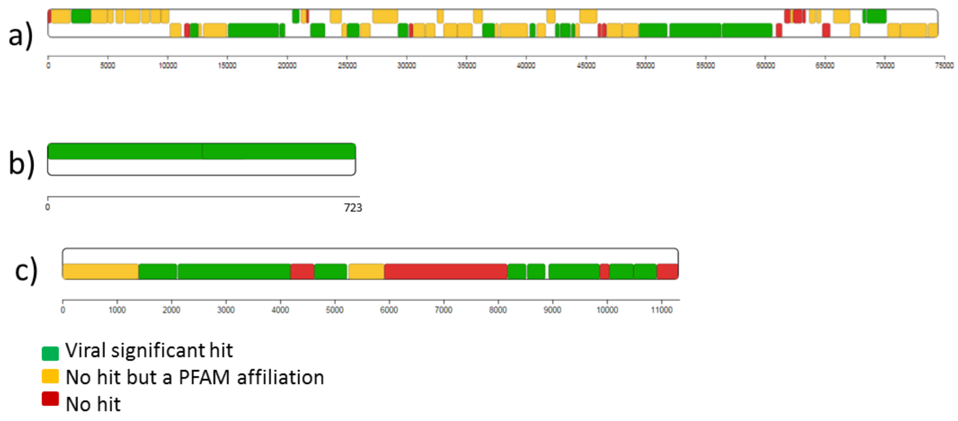

Supplementary Figure 3: Annotation of viral contigs showing positive viral annotation for a) contig 4 S1a; b) contig 3438 S1a, c) contig 64 S1a
